# Survival status and predictors of mortality among cervical cancer patients at oncologic centers in Addis Ababa, Ethiopia: a follow up study

**DOI:** 10.1186/s12885-024-12518-w

**Published:** 2024-06-20

**Authors:** Samuel Dessu Sifer

**Affiliations:** Department of Public Health, Yekatit 12 Hospital Medical College, Addis Ababa, Ethiopia

**Keywords:** Survival status, Predictors, Cervical cancer, Oncologic centers, Addis Ababa

## Abstract

**Background:**

Cervical cancer (CC) ranks as the third most commonly diagnosed cancer and the fourth leading cause of cancer-related deaths among women globally. In Addis Ababa, there is a shortage of available evidence concerning the phenomenon of survival time and its predictors among women diagnosed with CC. Therefore, this study aimed to assess the survival status and predictors of mortality among CC patients at oncologic centers in Addis Ababa, Ethiopia.

**Methods:**

A facility-based retrospective cohort study was conducted among records of women with cervical cancer enrolled from the 1st of January 2017 to the 30th of December 2022 among 252 cervical cancer patients. Data were collected using a pretested, structured data collection checklist by trained data collectors. The Kaplan–Meier survival curve was used to estimate the survival time of the respondents. The Cox multivariable regression model was carried out to identify predictors of CC. Variables with *P*-value < 0.05 in multivariable analysis were declared as statistically significant.

**Results:**

The cumulative proportion of surviving at the end of the 10th and 20th month was 99.6% (95%CI: 97.02, 99.94) and 96.99% (95%CI: 93.41, 98.64), respectively. Similarly, it was 92.67% (95%CI: 87.65, 95.70), 85.9% (95%CI: 78.68, 90.94), 68.0% (95%CI: 57.14, 76.66) and 18.27% (8.38, 31.16) at the end of 30th, 40th, 50th and 60th monthly respectively. The overall median survival time was 54 months (95%CI: 52.6, 55.4). The incidence of death among a cohort of women with CC was 7.34 per 1000 person months. Being anemic (AHR: 4.77; 95%CI: 1.93, 11.77; *P*-value: 0.001), took a single cancer treatment (AHR: 1.92; 95%CI: 1.01, 3.64; *P*-value: 0.046) and HIV sero status positive (AHR: 2.05; 95%CI: 1.01, 4.19; *P*-value: 0.048) were statistically significant in multivariable cox proportional hazard model.

**Conclusion and recommendation:**

Anemia, treatment initiation and HIV-sero status were independent predictors of mortality among women admitted with CC. It is imperative to enhance early screening initiatives and treatment resources for CC, alongside fostering public awareness through collaboration with various media outlets concerning preventive measures, screening procedures, and treatment alternatives for CC.

## Background

Cervical cancer (CC) is a significant contributor to female mortality worldwide [1]. It ranks as the third most commonly diagnosed cancer and the fourth leading cause of cancer-related deaths among women globally [1, 2]. In 2020, there were 604,127 new cases and 341,831 deaths attributed to cervical cancer worldwide [2]. Alarmingly, over 85% of these cases and deaths occurred in low-and middle-income countries (LMICs) [1, 2]. Eastern, Western, and Southern Africa are the regions with the highest incidence rates of cervical cancer globally [1, 2].

In 2020, Sub-Saharan Africa (SSA) reported 54,560 new cases of cervical cancer, resulting in 36,497 deaths [2]. Similarly, Ethiopia recorded 7,445 new cases of cervical cancer and 5,338 deaths during the same year [2]. Studies have highlighted a prevalent trend of late-stage disease reporting, with the highest incidence of cervical cancer occurring between the ages of 55 and 59 [3–5]. Risk factors identified in other studies include early marriage, multiple sexual partners, multiple pregnancies, oral contraceptive use, and lack of awareness [2, 3]. A study in Uganda revealed that 99% of women were aware of cervical cancer, with 63% associating family planning as a potential cause [4]. Moreover, 85% recognized inter-menstrual bleeding as a symptom of cervical cancer [5, 6].

Data from the radiotherapy center at Tikur Anbesa Specialized Hospital (TASH) indicate that cervical cancer ranks as the second most prevalent female cancer among patients attending the oncology center [5]. Additionally, one in four women in Ethiopia initiates sexual intercourse before the age of 15 [6], potentially increasing the risk of contracting the Human Papillomavirus (HPV), which is the causative agent of cervical cancer [7]. Moreover, studies have suggested that the level of cervical cancer screening in Ethiopia is less than 10% [7, 8].

Even though, there are studies conducted in Ethiopia to assess the prevalence of death and its associated factors among women with CC [5, 7, 8, 9], their survival time since the initiation of cancer treatment is not adequately investigated. Hence, the time to death among women with CC among women in Ethiopia is not well known. Therefore, this study aimed to assess the survival status and predictors of mortality among cervical cancer patients at oncologic centers in Addis Ababa, Ethiopia.

## Methods and materials

### Study area and period

A facility-based retrospective cohort study was conducted at oncologic centers in Addis Ababa among records of women with cervical cancer enrolled from the 1st of January 2017 to the 30th of December 2022. The study encompassed three selected governmental hospitals (Tikur Anbessa Specialized Hospital, St. Paul Hospital Millennium Medical College and Yakatit 12 Hospital Medical College based on the presence and absence of oncology centers.

### Populations

All women admitted with cervical cancer at oncologic centers in Addis Ababa were considered as source populations and all the randomly selected records of women admitted with CC at oncologic centers in Addis Ababa were considered as study populations.

It was an open cohort study, allowing records of cervical cancer patients to freely enter and exit the cohort at any point during the study period. The starting point was from admission to the oncologic centers within the study period and the end point was either death or recovery, loss to follow up and follow up time was completed without outcome happened. In addition, since it was one-arm study only those women admitted with cervical cancer to oncologic centers from within the study period were selected and compared between died and survived during analysis.

### Eligibility criteria

All the records of women admitted with CC at oncologic centers enrolled from within the study period were included in the study and all the records of CC diagnosed women which lack the enrollment period, status, dead with emergency causes (other than comorbidity) and the patient discharged without knowing the medical status were excluded from the study.

### Sample size determination and sampling procedure

The required sample size for the study was determined using a double population proportion formula (file:///C:/Users/User/AppData/Local/Temp/Rar$EXa10460.49237/OpenEpi/Menu/OE_Menu.htm) in considering the following assumptions: power: 80, Ratio of sample size, Unexposed/Exposed: 1, Percent of Unexposed with Outcome for a variable FIGO stage 2: 87%, Percent of Exposed with Outcome: 97%, AHR: 4.6 and which yields sample size became of 272. The total number of cervical cancer records across the three hospitals-in the study period was 1916. Since the total number of records was below 10,000, a correction formula was used; which is n_i_= $$\frac{n}{1+\frac{n}{N}}$$, through instituting the numbers with the formula,

$$\frac{272}{1+\frac{272}{2416}} = 244.$$

Adding 10% for the incomplete record (non-response rate) yields a final sample size of 268.

All the oncologic centers found in Addis Ababa were selected. After that, all records of women with cervical cancer in three selected hospitals from January 1st, 2017, to December 30th, 2022 were identified from the database by their medical registration numbers. Then, random medical registration numbers were chosen using a computer-generated approach. For each oncologic center, a sampling frame was identified and the total sample size was allocated for each hospital proportionally. Finally, simple random sampling method was employed to select individual participants from each hospital.

### Variables

The dependent variable was time to death and the independent variables were socio demographic and individual level factors (marital status, education, residential address, age at diagnosis, substance use, number of children, region, occupation, religion), pathological and clinical factors (stage at presentation, histology type, baseline anemia, comorbidity, types of co morbidity) and treatment related factors (chemotherapy, radiation, surgery, aim of radiotherapy, combination of treatments modalities) and variables in relation to clinical stage and treatment were clinical extent of disease classified according to Federation of Gynecology and Obstetrics (FIGO) system (stage I, stage II, stage III, stage IV, and unknown) performance status (WHO) before treatment classified as active, not active and bedridden (WHO performance status) [4].

### Operational and term definition

#### Censored

Any woman admitted with cervical cancer that is loss to follow up, death status not known, discharged alive, and did not develop the outcome up to the end of the study period is considered as censored [5].

#### Overall survival

is calculated from the date of diagnosis to the moment of death or last contact [5].

#### Comorbidity

a Cervical cancer women record contain at least one of the chronic diseases (myocardial infarction, congestive heart failure, peripheral vascular disease, cerebrovascular disease, dementia, chronic pulmonary disease, connective tissue disease, ulcer disease, mild liver disease, diabetes, hemiplegia, moderate or several renal disease, diabetes with end organ damage, any tumor, leukemia, lymphoma, moderate or severe liver disease, metastatic solid tumor, AIDS) [7].

#### Time to event

is described from the time of diagnosis till the date of an appearance of death.

### Data collection procedures

Data collection format/check list was developed for collecting data from study participant’s medical chart anonymously from the selected health facilities. Data collection format has been prepared in English version and collected by trained nurses. All study participants’ records were selected based on the eligibility criteria. All medical records of women with cervical cancer who meet the inclusion criteria from January 1st, 2017 to December 30th, 2022 in the selected oncologic centers were retrospectively reviewed. A medical death certificate was reviewed in the hospital to confirm death.

### Data quality control

Data quality was maintained by designing an appropriate data abstraction tool. A pretest was conducted on 5% of the sample size at Zewuditu Memorial Hospital two weeks before the actual study, using an organized checklist to verify commonly recorded variables in patients’ medical records. The data collector and supervisor were experienced MSc nurses with expertise in caring for women with cervical cancer and were currently employed in this unit. They received one day of effective training. Supervisors and investigators closely monitored the data collection process to ensure high-quality data. Data completeness was checked daily, and any encountered challenges were addressed promptly. Additionally, during data administration, storage, and analysis, both the supervisor and investigator double-checked all obtained data for completeness and consistency.

### Data processing and analysis

Data were entered using Epi-Data version 4.6 and analyzed using SPSS version 26. The study participant’s status was categorized into survived or dead. Incidence rate (IR) was determined. The Kaplan-Meier survival curve was employed to estimate the mean survival time and cumulative probability of survival. Log-rank tests were performed to compare survival curves post-admission among patient groups categorized by predictors. Additionally, for each explanatory variable, a bivariate Cox-proportional hazards regression model was examined. Hazard ratios with 95% confidence intervals and *p*-values were utilized to assess the strength of the association and determine the statistical significance of the results. Variables with *P*-value less than 0.05 in multivariable Cox proportional hazard were considered statistically significant.

## Results

### Socio demographic and behavioral characteristics

This study was conducted with a total of 252 study participant records, which yields a response rate of 94%. Nearly one-fourth of the dead women (33, 27.0%) were aged 30–60 years, while 89 (73.0%) of the survivors fell within the same age category as the dead. Regarding the place of residence, 27 (16.1%) of the dead and 141 (83.9%) of the survivors were urban residents. Similarly, 10 (15.9%) of the dead and 54 (84.4%) of the survivors had studied at the college level or above. Moreover, 24 (24.0%) of the dead and 76 (76.0%) of the survivors were government employees. Additionally, 37 (27.0%) of the dead and 100 (73.0%) of the survivors were single (Table 1).

**Table 1:** Socio demographic characteristics of women with CC at oncologic centers in Addis Ababa, Ethiopia

Variables	Category	Status	
		Died [n (%)]	Survived [n (%)]
Age (in years)	Below 30	27 (22.9%)	91 (77.1%)
	30–60	33 (27.0%)	89 (73.0%)
	More than 60	0 (0%)	12 (100.0%)
Place of residence	Urban	27 (16.1%)	141 (83.9%)
	Rural	33 (39.3%)	51 (60.7%)
Level of education	Unable to read and write	14 (46.7%)	16 (53.3%)
	Only read and write	13 (28.9%)	32 (71.1%)
	Primary	12 (23.5%)	39 (76.5%)
	Secondary	11 (17.7%)	51 (82.3%)
	College and above	10 (15.6%)	54 (84.4%)
Occupation	Government employee	24 (24.0%)	76 (76.0%)
	Private employee	25 (23.4%)	82 (76.6%)
	Merchants	3 (14.3%)	18 (85.7%)
	Others	8 (33.3%)	16 (66.7%)
Marital status	Married	21 (20.0%)	84 (80.0%)
	Single	37 (27.0%)	100 (73.0%)
	Divorced	2 (25.0%)	6 (75.0%)
	Widowed	0 (0.0%)	2 (100.0%)
Substance use	Yes	24 (42.1%)	33 (57.9%)
	No	36 (18.5%)	159 (81.5%)
Alcohol consumption	Yes	31 (24.6%)	95 (75.4%)
	No	29 (23.0%)	97 (77.0%)

### Gynecologic and related characteristics

The majority of the dead (37, 19.9%) and 149 (80.1%) of the survivors had no history of sexually transmitted infections. One-fifth (12, 11.8%) of the dead and 90 (88.2%) of the survivors reported a history of contraceptive use. Furthermore, 17 (45.9%) of the dead and 20 (54.1%) of the survivors had a history of vaginal bleeding (Table 2).

**Table 2:** Gynecologic and related characteristics of the women with CC at oncologic centers in Addis Ababa, Ethiopia

Variables	Category	Status	
		Died [n (%)]	Survived [n (%)]
Sexually transmitted infections	Yes	23 (34.8%)	43 (65.2%)
	No	37 (19.9%)	149 (80.1%)
Contraceptive use	Yes	12 (11.8%)	90 (88.2%)
	No	48 (32.0%)	102 (68.0%)
Vaginal bleeding	Yes	17 (45.9%)	20 (54.1%)
	No	43 (20.0%)	172 (76.2%)

### Medical and related characteristics

Of the total who survived, more than half (20, 54.1%) had utilized traditional medicine, whereas 17 (45.9%) of the deceased had done so. Regarding the cancer stage, nearly half of the deceased (25, 49%) and 26 (51.0%) of the survivors had stage IV cancer, respectively. Similarly, 36 (39.6%) of the dead and 55 (60.4%) of the survived were HIV seropositive. Anemia was prevalent among women, with 49 (67.1%) survived and 24 (32.9%) dead individuals having developed it. Moreover, 22 (37.9%) of the dead and 36 (62.1%) of the survived had developed adenocarcinoma (Table 3).

**Table 3:** Medical and related characteristics of the women with CC at oncologic centers in Addis Ababa, Ethiopia

Variables	Category	Status	
		Died [*n* (%)]	Survived [*n* (%)]
Previous practice of cervical cancer screening	Yes	29 (45.3%)	35 (54.7%)
	No	31 (16.5%)	157 (83.5%)
Traditional medicine utilization	Yes	17 (45.9%)	20 (54.1%)
	No	43 (20.0%)	172 (80.0%)
Comorbid health problem	Yes	23 (40.4%)	34 (59.6%)
	No	37 (19.0%)	158 (81.0%)
Stage of cancer	Stage I	10 (9.6%)	94 (90.4%)
	Stage II	12 (24.5%)	37 (75.5%)
	Stage III	13 (27.1%)	35 (72.9%)
	Stage IV	25 (49.0%)	26 (51.0%)
Anemia	Yes	49 (67.1%)	24 (32.9%)
	No	11 (6.1%)	168 (93.9%)
Treatment	At least two of surgery, chemotherapy and radiotherapy	35 (17.0%)	171 (83.0%)
	Either surgery or chemotherapy or radiotherapy	25 (54.3%)	21 (45.7%)
Adenocarcinoma	Yes	22 (37.9%)	36 (62.1%)
	No	38 (19.6%)	156 (80.4%)
HIV sero status	Positive	36 (39.6%)	55 (60.4%)
	Negative	24 (14.9%)	137 (85.1%)

### Survival time to death among women with cervical cancer

**Fig. 1:**
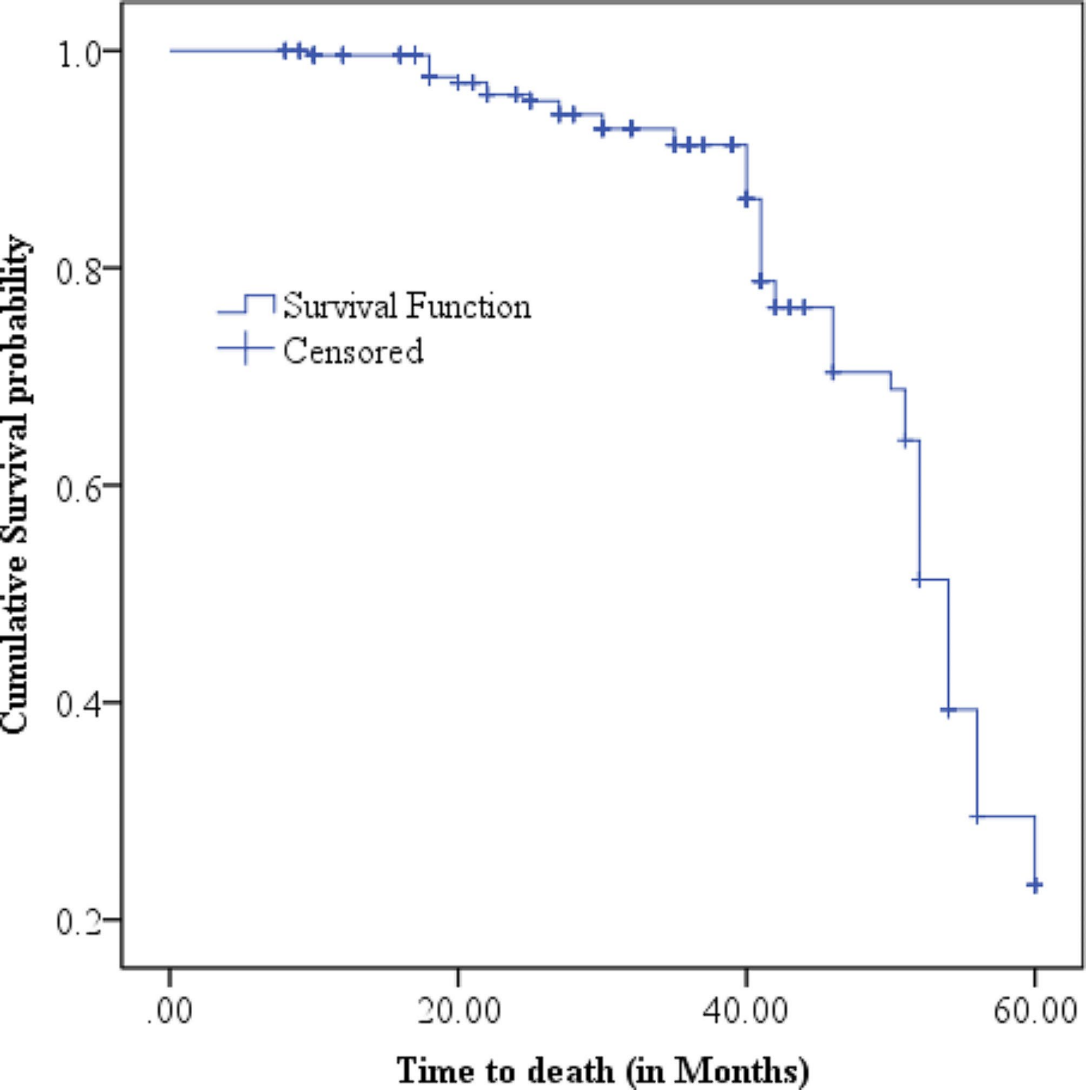
The Kaplan Maier estimate of time to death among women with CC at oncologic centers in Addis Ababa, Ethiopia

The cumulative proportion of survival at the end of the 10th and 20th month was 99.6% (95% CI: 97.02, 99.94) and 96.99% (95% CI: 93.41, 98.64), respectively. Similarly, it was 92.67% (95% CI: 87.65, 95.70), 85.9% (95% CI: 78.68, 90.94), 68.0% (95% CI: 57.14, 76.66), and 18.27% (95% CI: 8.38, 31.16) at the end of the 30th, 40th, 50th, and 60th month, respectively. The overall median survival time was 54 months (95% CI: 52.6, 55.4). In this study, the total person-time of observation was 8167 months, and among the cohort of study subjects, 60 were dead. Therefore, the incidence rate of death among a cohort of women with CC was 7.34 per 1000 person-months (60 deaths or events / 8167 patient-months) (Fig. 1).

The cumulative proportion of survival at the end of the 20th, 40th, and 60th month among those with anemia was 92.48% (95% CI: 82.86, 96.80), 70.15% (95% CI: 56.63, 80.16), and 41.5% (95% CI: 0.61, 13.77), respectively. In contrast, among those without anemia within the same period, it was 99.23% (95% CI: 94.65, 99.89), 97.2% (95% CI: 87.81, 99.38), and 56.35% (95% CI: 32.31, 74.74), respectively.

**Fig. 2:**
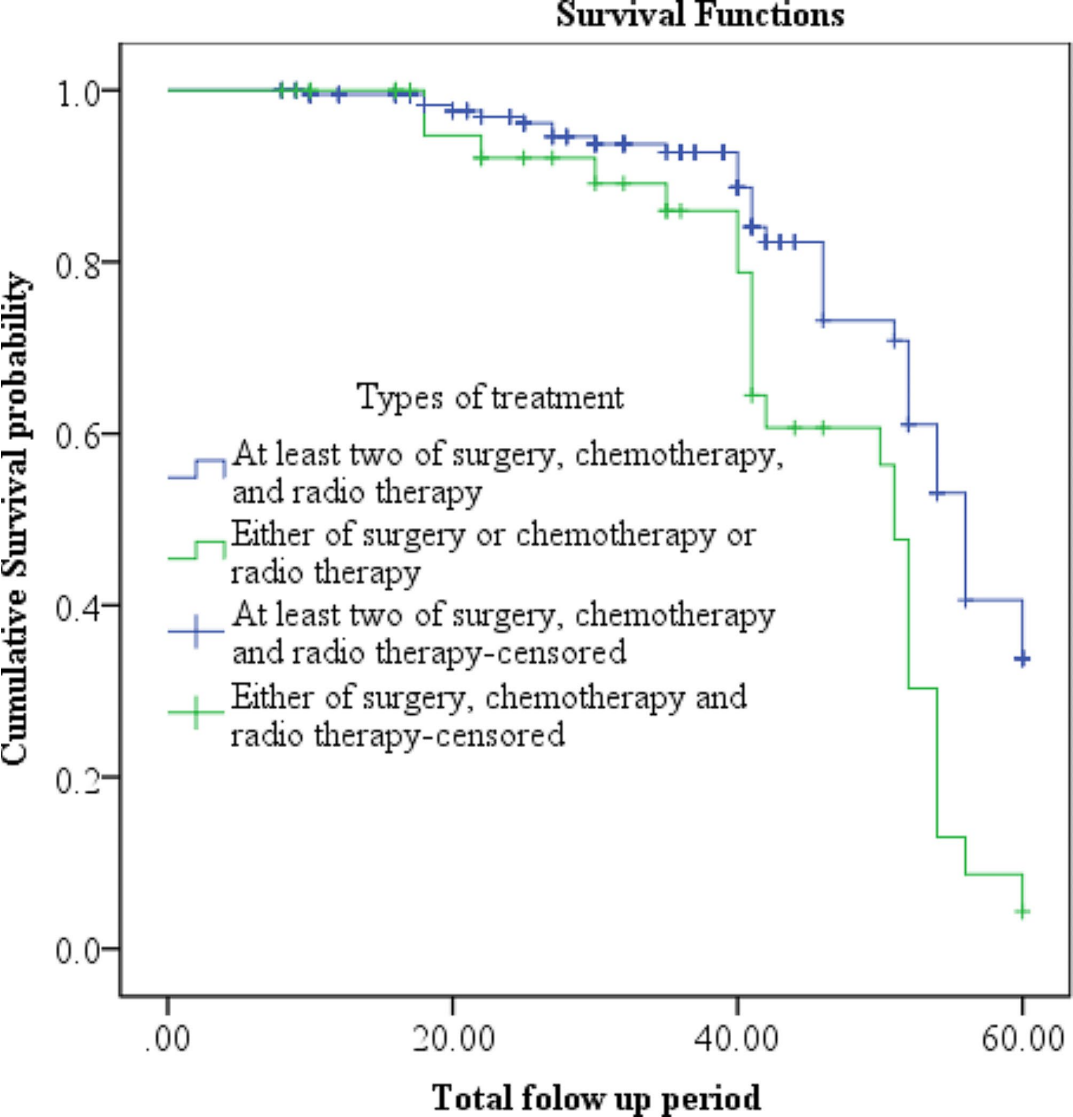
Effect of cancer treatment on the survival status of the CC patients at oncologic centers in Addis Ababa, Ethiopia

Consistently, the cumulative proportion of survival at the end of the 20th, 40th, and 60th month among those who underwent at least two of surgery, chemotherapy, and radiotherapy was 97.54% (95% CI: 93.54, 99.07), 88.31% (95% CI: 80.03, 93.29), and 27.96% (95% CI: 12.74, 45.47), respectively. In comparison, among those who underwent either surgery or chemotherapy or radiotherapy within the same period, it was 92.03% (95% CI: 77.28, 97.36), 78.57% (95% CI: 59.87, 89.28), and 2.87% (95% CI: 0.06, 18.28), respectively (Fig. 2).

**Fig. 3:**
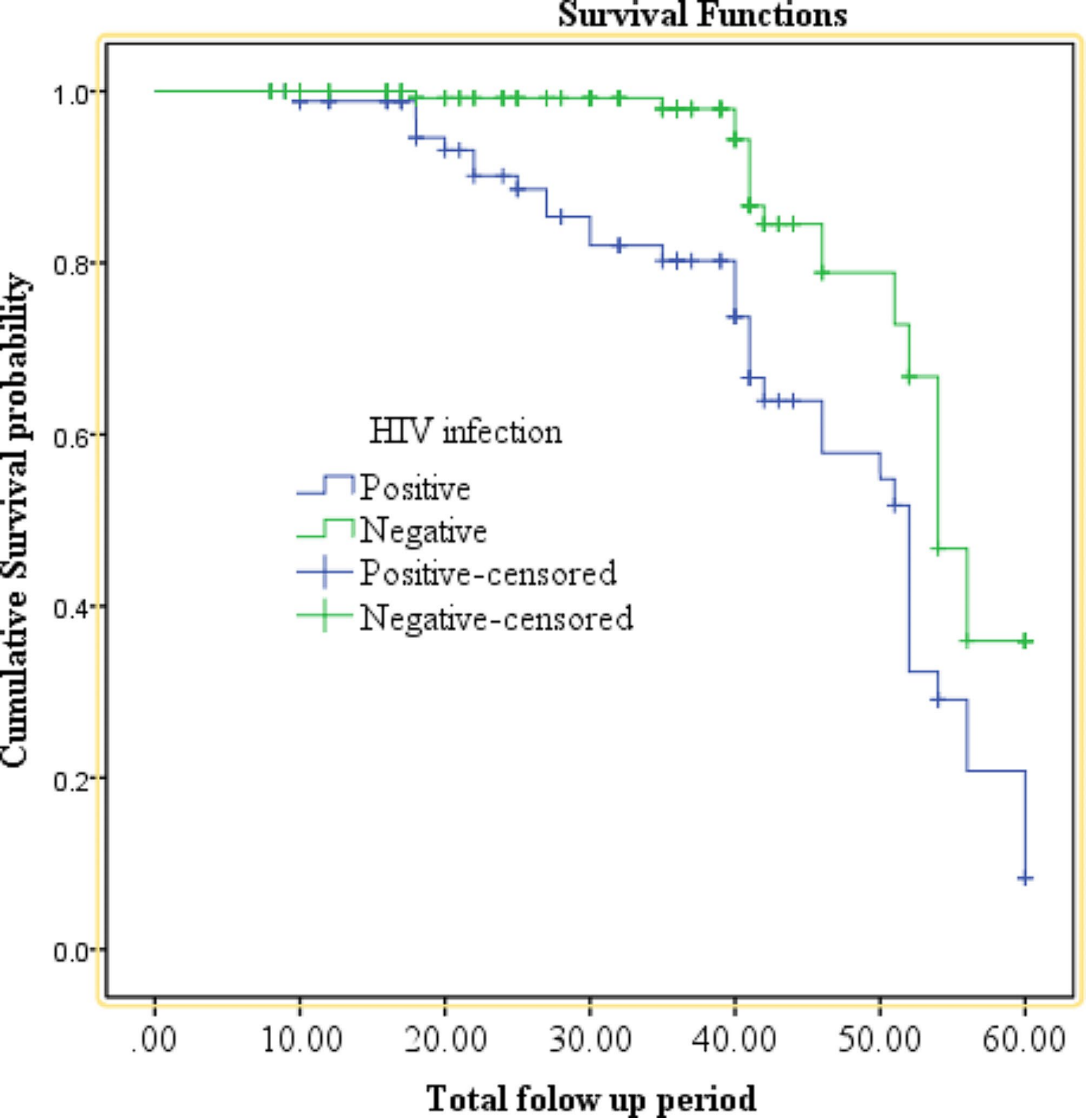
The effect of HIV infection on the survival status of the CC patients at oncologic centers in Addis Ababa, Ethiopia

In addition, the cumulative proportion of survival at the end of the 20th, 40th, and 60th month among HIV seropositive individuals was 93.05% (95% CI: 84.06, 97.06), 73.32% (95% CI: 59.68, 82.97), and 5.06% (95% CI: 0.04, 20.00), respectively. Meanwhile, among those who were HIV seronegative, the cumulative proportion of survival was 99.2% (95% CI: 94.46, 99.89), 94.19% (95% CI: 84.62, 97.88), and 34.62% (95% CI: 18.67, 51.15) at the end of the 20th, 40th, and 60th month, respectively (Fig. 3).

### Predictors of mortality among women admitted with cervical cancer

Place of residence, level of education, STI, contraceptive utilization, vaginal bleeding, substance use, comorbidity, stage of cancer, anemia, treatment initiation, and having adenocarcinoma were variables considered for the multivariable analysis. Among these candidate variables, anemia, treatment initiation, and HIV serostatus were statistically significant in the multivariable Cox proportional hazards model.

Women with cervical cancer who have anemia have a 4.77 times higher risk of mortality compared to those without anemia (AHR: 4.77; 95% CI: 1.93, 11.77; *P*-value: 0.001). The risk of mortality among women with cervical cancer who received only one form of treatment was 1.92 times higher compared to those who received at least two treatments (surgery, radiation, and chemotherapy) (AHR: 1.92; 95% CI: 1.01, 3.64; *P*-value: 0.046). Additionally, the risk of mortality among HIV-positive women admitted with cervical cancer was 2.05 times higher compared to those who were HIV-negative (AHR: 2.05; 95% CI: 1.01, 4.19; *P*-value: 0.048) (Table 4).

**Table 4:** Predictors of mortality among women with CC at oncologic centers in Addis Ababa, Ethiopia

Variables	Category	Status		CHR (95%CI)	AHR (95%CI)	*P*-value
		Died	Survived			
Place of residence	Urban	27	141	1	1	
	Rural	33	51	2.07 (1.24, 3.45)*	1.71 (0.79, 3.71)	0.173
Level of education	Unable to read and write	14	16	1.44 (0.63, 3.26)	0.45 (0.14, 1.38)	0.161
	Only read and write	13	32	1.61 (0.70, 3.67)	0.85 (0.32, 2.28)	0.743
	Primary	12	39	1.89 (0.81, 4.37)*	0.51 (0.16, 1.61)	0.247
	Secondary	11	51	0.99 (0.42, 2.35)	0.63 (0.22, 1.79)	0.385
	College and above	10	54	1	1	
STI	Yes	23	43	1.48 (0.88, 2.48)*	2.02 (0.78, 5.23)	0.149
	No	37	149	1	1	
Contraceptive utilization	Yes	12	90	1	1	
	No	48	102	2.83 (1.49, 5.35)*	1.13 (0.46, 2.78)	0.797
Vaginal bleeding	Yes	17	20	1.41 (0.79, 2.48)*	0.83 (0.37, 1.89)	0.665
	No	43	172	1	1	
Substance use	Yes	24	33	1.97 (1.17, 3.12)*	1.45 (0.75, 2.80)	0.271
	No	36	159	1	1	
Comorbidity	Yes	23	34	1.73 (1.03, 2.91)*	0.99 (0.39, 2.48)	0.984
	No	37	158	1	1	
Stage of cancer	Stage I	10	94	1	1	
	Stage II	12	37	1.81 (0.78, 4.22)*	0.77 (0.28, 2.17)	0.622
	Stage III	13	35	2.24 (0.98, 5.12)*	1.47 (0.54, 3.98)	0.447
	Stage IV	25	26	2.31 (1.11, 4.83)*	0.66 (0.25, 1.76)	0.405
Anemia	Yes	49	24	5.69 (2.95, 11.00)*	4.77 (1.93, 11.77)**	**0.001**
	No	11	168	1	1	
Treatment	At least two: surgery, chemotherapy, radiotherapy	35	171	1	1	
	Either surgery or chemotherapy or radiotherapy	25	21	2.34 (1.39, 3.92)*	1.92 (1.01, 3.64)**	**0.046**
HIV sero status	Positive	36	55	2.51 (1.49, 4.21)*	2.05 (1.01, 4.19)**	**0.048**
	Negative	24	137	1		

*indicates variables having *P*-value < 0.25 in bivariate analysis and ** indicates variables having *P*-value < 0.05 in multivariable Cox Proportional Hazard Model

## Discussion

The objective of this study was to assess the survival outcomes and predictors of mortality among women diagnosed with cervical cancer at oncologic centers in Addis Ababa. The cumulative proportion of survival at the end of the 10th and 20th month was 99.6% (95% CI: 97.02, 99.94) and 96.99% (95% CI: 93.41, 98.64), respectively. Similarly, it was 92.67% (95% CI: 87.65, 95.70), 85.9% (95% CI: 78.68, 90.94), 68.0% (95% CI: 57.14, 76.66), and 18.27% (95% CI: 8.38, 31.16) at the end of the 30th, 40th, 50th, and 60th month, respectively. These rates are lower compared to a study conducted in Malaysia, which reported survival rates of 94.1%, 79.3%, and 71.1% at one, three, and five years, respectively [10]. Differences in findings might stem from variations in sample sizes and study durations. Changes in treatment methodologies, healthcare policies related to cervical cancer, and the availability of infrastructure for early detection and treatment may have evolved over different time frames.

The incidence rate of mortality among women diagnosed with cervical cancer stood at 7.34 per 1,000 person-months. This rate surpasses the reported incidence of cervical cancer mortality in Sub-Saharan Africa [11]. The observed gap could stem from differences in study duration, the stage at which cancer was diagnosed, delays in accessing treatment after diagnosis, and variances in the quality of cancer care services [8, 12].

Anemia was found to be an independent predictor of mortality among women with cervical cancer. The cumulative proportion of survival at the end of the 60th month was 41.5% for those with anemia, compared to 56.35% for those without anemia within the same period. This result is supported by studies conducted in Nigeria [13], Ethiopia [14, 15], and at Tikur Anbesa Specialized Hospital in Addis Ababa, Ethiopia [16]. This agreement might be based on the observation that reduced hemoglobin levels in the bloodstream can cause oxygen deprivation in both cancerous and healthy cells, ultimately resulting in unintended cell death [17].

Scientifically, treatments are acknowledged to prolong the survival duration of patients. Among women diagnosed with cervical cancer, those who received only one form of treatment had a higher risk of mortality compared to those who underwent at least two treatments, such as surgery, chemotherapy, or radiotherapy. This observation is supported by various studies in the literature [12, 17, 18].

The cumulative proportion of survival at the end of the 60th month of follow-up was 27.96% among those who received at least two treatments (surgery, chemotherapy, or radiotherapy), compared to 2.87% among those who received only one form of treatment within the same period. Unlike similar studies, our research did not identify the specific treatment that contributed to enhancing the survival of cervical cancer patients (CCPs) [19]. However, this Kaplan-Meier analysis revealed a significant disparity in the median survival time between CC patients who underwent surgery and those who did not. A similar outcome was documented in a study conducted at TASH five years prior [20].

In this study, among participants for whom HIV status was documented on their patient cards, those identified as HIV-positive exhibited mortality risk approximately twice as high as those who were HIV-negative. This finding was consistent with a study conducted at Tikur Anbessa Specialized Hospital [21]. A plausible scientific explanation is that HIV diminishes the efficacy of cellular immune responses, affecting the capacity to sustain oncologic remission [22]. Furthermore, HIV might lower tolerance to chemotherapy and radiation therapy, and increase the prevalence of anemia in HIV-infected women, consequently undermining the effectiveness of radiation treatment [22–24]. This consistency is evidenced in studies conducted in Brazil [25], Botswana [26], and Kenya [23].

### Limitations of the study

This study is limited by incomplete data on important variables such as nutritional status, age at sexual debut, the number of sexual partners, and type of STI, which could potentially introduce confounding factors into the results. Moreover, relying on secondary data means that the reliability of the information is contingent upon the accuracy and comprehensiveness of the cancer patient records. Additionally, inadequate information regarding the cause of death impedes the accurate identification of the underlying causes of death for patients listed as deceased.

## Conclusion

The death rate of cervical cancer (CC) patients was found to be high compared to previously conducted studies. Significant differences were observed in the median survival time between categories of covariates such as age, stages of cervical cancer, received treatment, surgery, and HIV status. Anemia, the number of cancer treatments, and HIV serostatus were independent predictors of mortality among women admitted with cervical cancer.

It is imperative to enhance early screening initiatives and treatment resources for CC, alongside fostering public awareness through collaboration with various media outlets concerning preventive measures, screening procedures, and treatment alternatives for CC. Furthermore, healthcare professionals must promptly commence treatment for all CC patients to enhance their survival rates. Strengthening routine CC screening programs, particularly targeting high-risk groups such as women living with HIV, is crucial. Additionally, prospective studies are recommended to comprehensively address factors such as nutritional status and sexual and related factors.

## Data Availability

No datasets were generated or analysed during the current study.

## Abbreviations

CA: Cervical Cancer
CIN: Cervical Intra-Epithelial Neoplasia
HPV: Human Papilloma virus
LSTF: Lost to Follow up
LMICs: Low-and-middle income countries
WHO: World Health Organization
